# Effect of ciclosporin on safety, lymphocyte kinetics and left ventricular remodelling in acute myocardial infarction

**DOI:** 10.1111/bcp.14252

**Published:** 2020-03-11

**Authors:** Suzanne Cormack, Ashfaq Mohammed, Pedram Panahi, Rajiv Das, Alison J. Steel, Thomas Chadwick, Andrew Bryant, Mohaned Egred, Konstantinos Stellos, Ioakim Spyridopoulos, the CAPRI investigators

**Affiliations:** ^1^ Freeman Hospital Newcastle upon Tyne UK; ^2^ Translational and Clinical Research Institute, Faculty of Medical Sciences Newcastle University UK; ^3^ Faculty of Health and Life Sciences Northumbria University UK; ^4^ Newcastle Clinical Trials Unit, Faculty of Medical Sciences Newcastle University UK; ^5^ Population Health Sciences Institute Newcastle University UK

**Keywords:** acute myocardial infarction, cardiac MRI, ciclosporin, left ventricular function, reperfusion injury, T‐lymphocytes

## Abstract

**Aims:**

Following a favourable pilot trial using a single bolus of ciclosporin, it has been unclear why 2 large studies (CYCLE and CIRCUS) failed to prevent reperfusion injury and reduce infarct size in STEMI (ST elevation myocardial infarction). The purpose of this study was to assess the effect of ciclosporin on myocardial injury, left ventricular remodelling and lymphocyte kinetics in patients with acute STEMI undergoing primary percutaneous coronary intervention.

**Methods:**

In this double‐blind, single centre trial, we randomly assigned 52 acute STEMI patients with an onset of pain of <6 hours and blocked culprit artery to a single bolus of ciclosporin (*n* = 26) or placebo (n = 26, control group) prior to reperfusion by stent percutaneous coronary intervention. The primary endpoint was infarct size at 12 weeks.

**Results:**

Mean infarct size at 12 weeks was identical in both groups (9.1% [standard deviation= 7.0] *vs* 9.1% [standard deviation = 7.0], *P* = .99; 95% confidence interval for difference: −4.0 to 4.1). CD3 T‐lymphocytes dropped to similar levels at 90 minutes (867 *vs* 852 cells/μL, control *vs* ciclosporin) and increased to 1454 *vs* 1650 cells/μL at 24 hours.

**Conclusion:**

In our pilot trial, a single ciclosporin bolus did not affect infarct size or left ventricular remodelling, matching the results from CYCLE and CIRCUS. Our study suggests that ciclosporin does either not reach ischaemic cardiomyocytes, or requires earlier application during first medical contact. Finally, 1 bolus of ciclosporin is not sufficient to inhibit CD4 T‐lymphocyte proliferation during remodelling. We therefore believe that further studies are warranted.

(Evaluating the effectiveness of intravenous Ciclosporin on reducing reperfusion injury in pAtients undergoing PRImary percutaneous coronary intervention [CAPRI]; NCT02390674)

**What is already known about this subject**

Mitochondria are thought to be the critical mediator of ischaemia reperfusion injury, and drugs that target the mitochondrial permeability transition pore have been successful tested in a multitude of preclinical models. Two clinical trials could not demonstrate a beneficial effect of the mitochondrial permeability transition pore inhibitor ciclosporin bolus prior to myocardial reperfusion, but the mechanism is unclear.

**What this study adds**

Intravenous administration of ciclosporin immediately before primary coronary angioplasty is safe, but does not affect myocardial injury, ventricular remodelling or leucocyte kinetics. Serum levels of ciclosporin during reperfusion were 10‐fold higher than established therapeutic levels for immunosuppression, suggesting that either (i) ciclosporin does not reach ischaemic cardiomyocytes, or (ii) ciclosporin needs to be given at an earlier timepoint during myocardial ischaemia (e.g. before transfer for primary percutaneous coronary intervention). Also, 1 bolus of ciclosporin is not sufficient to inhibit CD4 T‐lymphocyte proliferation during remodelling.

## 1 INTRODUCTION

Primary percutaneous coronary intervention (PPCI) has improved outcomes for patients with ST‐segment myocardial infarction (STEMI) dramatically over the past years. In‐hospital mortality rates in high volume centres have dropped <5%, owing to rapid reperfusion, optimal antiplatelet therapy and latest‐generation stents. However, 5‐year mortality rate in all‐comers with large infarcts undergoing PPCI has been around 20% in our centre,^1^ supporting the fact that adverse remodelling and potential progression to heart failure still present an unfulfilled clinical need.^2^ In preclinical models of myocardial infarction, reperfusion itself leads to additional damage known as myocardial ischaemia/reperfusion injury (IRI). Since mitochondria are thought to be the critical mediator of IRI,^3^ drugs that target the mitochondrial permeability transition pore (MPTP) have been tested in a multitude of animal models.^3^, ^4^, ^5^ A pilot trial by Piot and colleagues demonstrated a favourable effect of ciclosporin in patients on infarct size, estimated by the release of creatine kinase following reperfusion.^6^ In addition to its known immunosuppressive function, ciclosporin is also a potent inhibitor of the MPTP,^7^ which has demonstrated a beneficial effect on ischaemia/reperfusion injury under experimental conditions.^8^ However, 2 recent multicentre trials (published after the start of the CAPRI trial) using ciclosporin prior to PPCI have failed to demonstrate a beneficial effect.^9^, ^10^ It has been debated whether either preclinical models do not resemble the clinical situation, or whether there have been inadequacies in the trial design and, more importantly, whether cardioprotective therapies should continue to be pursued at all.^11^ A significant drawback of the negative clinical trials targeting IRI has been the lack of mechanistic insight into their failure. Ciclosporin is widely used as an immunosuppressive drug to prevent organ transplant rejection.^12^, ^13^, ^14^ Through its intracellular binding to cyclophylin it inhibits calcineurin, a serine/threonine phosphatase, and thereby inhibits various T‐cell responses,^14^ most importantly T‐lymphocyte proliferation.^15^ Our recently published data also suggests depletion of circulating T‐lymphocytes in STEMI patients immediately following PPCI, consistent with an early participation of T‐lymphocytes in human myocardial I‐R injury,^16^ making lymphocytes another pharmacological target in the treatment of STEMI.

The aim of our pilot trial was to investigate the effect of ciclosporin on IRI and concomitantly achieve mechanistic insight by studying its biological effects on the adaptive immune response as a proximal readout. To avoid design‐specific flaws, the CAPRI trial was set up as a single‐centre, double‐blinded and randomized trial using state of the art cardiac magnetic resonance imaging (MRI) to assess infarct size, microvascular obstruction and cardiac remodelling, confirm ciclosporin blood level kinetics, and finally quantify lymphocyte subset dynamics over 2 weeks from fresh blood samples. The results of CAPRI should indicate why previous studies might have yielded a negative result and provide information on potential targets for future clinical trials.

## 2 METHODS

### 2.1 (please see online Supplemental methods for additional information)

The CAPRI (‘Evaluating the effectiveness of intravenous **c**iclosporin on reducing reperfusion injury in p**a**tients undergoing **pri**mary percutaneous coronary intervention’) trial (www.clinicaltrials.gov NCT02390674, EudraCT number 2014–002628‐29) was a single‐centre, randomised, double‐blinded, controlled trial of ciclosporin *vs* placebo (saline) in patients with acute myocardial infarction. The trial was conducted in accordance with the principles of Good Clinical Practice and received a favourable ethical opinion from the National Research Ethics Committee North‐East—Newcastle and North Tyneside 2 (14/NE/1070) on 24 July 2014 and a clinical trial authorisation from the Medicines and Healthcare products Regulatory Agency (MHRA) on 9 September 2014. Recruitment occurred between March 2015 and November 2016. The trial was managed through the Newcastle Clinical Trials Unit together with an independent Trial Steering Committee and Data Monitoring and Ethics Committee (DMEC). Monitoring was performed by Newcastle Clinical Trials Unit and the trial was audited by the sponsor, Newcastle upon Tyne Hospitals NHS Foundation Trust and inspected by the MHRA. Following initial verbal consent in the presence of an independent witness in the cathlab, written informed consent was obtained from all patients after the PPCI procedure/intervention.

### 2.2 Study population

All participants had to present with an acute myocardial infarction (STEMI) and undergo PPCI, be aged ≥18 years, and present within 6 hours of the onset chest pain. The culprit coronary artery had to be a major coronary artery with a diameter of at least 3 mm and had to be proximally occluded (TIMI flow grade 0–1) at the time of admission coronary angiography.

Exclusion criteria for this trial were patients presenting with immunological disorders, cardiogenic shock, unconscious patients, evidence of coronary collaterals to the infarct area, open (TIMI>1) culprit coronary artery at the time of angiography, previous myocardial infarction or thrombolytic therapy, known renal or liver insufficiency, uncontrolled hypertension (>180/110 mmHg), female patients currently pregnant or women of childbearing age who were not using contraception (verbal diagnosis), or patients with contraindication to cardiac MRI.

### 2.3 Experimental protocol

Following angiography, participants were randomised in a 1:1 ratio to either ciclosporin or control (saline) using a blocked allocation (permuted random blocks of variable length) system. Randomisation included stratification by infarct location (anterior or nonanterior) and sex. This was implemented using Newcastle Clinical Trials Unit's online randomisation service. Ciclosporin (Sandimmun, Novartis) was given as an intravenous infusion dissolved in saline (maximum concentration 2.5 mg/mL) of 2.5 mg/kg of body weight (maximum total amount 250 mg) through a catheter positioned within a peripheral vein over 4 minutes. Trial medication was prepared by 2 unblinded, Good Clinical Practice‐trained research nurses according to local pharmacy policy. After successful infusion, the coronary wire was advanced and culprit lesion crossed, followed by revascularisation.

Primary outcome measure was infarct size at 12 weeks post‐PPCI as measured by cardiac MRI. Infarct size was calculated as the percent of infarcted myocardium per left ventricular (LV) mass.

Secondary outcome measures included cardiac MRI (end‐diastolic volume [EDV], end‐systolic volume [ESV], systolic volume, LV ejection fraction (LVEF), myocardial mass [diastole/systole]), late gadolinium enhancement outcomes at 2–7 days (baseline) and 12 weeks (microvascular obstruction at baseline only, infarct size, myocardial mass), change in T lymphocyte counts (B‐cells [CD19], NK‐cells, T‐cells [CD3] and CD4 and CD8 subtypes) relative to baseline at 5, 15, 30 and 90 minutes, and 24 hours postreperfusion. Additionally, we also reported the number of clinical events (all cause death, stroke or myocardial infarction) after 12 months. We additionally reported LV remodelling, kinetics of lymphocyte populations and additional T‐cell subpopulations in *posthoc* analyses.

### 2.4 Cardiac MRI

Cardiac MRI scans were obtained at 2–7 days as well as 12 weeks post‐myocardial infarction with a Siemens Avanto 1.5 Tesla MRI scanner, using a phased array body coil combined with a spine coil. Intravenous gadobutrol contrast (Gadovist, Bayer Schering Pharma AG, Berlin, Germany) was administered at a dose of 0.1 mmol/kg and, after 10 minutes, short axis end‐diastolic late gadolinium enhancement images were obtained. All analysis was performed using validated cardiac MRI analysis software (cvi42, Circle Cardiovascular Imaging Inc., Calgary, Canada) as previously described.^16^

### 2.5 Statistical analysis

The primary endpoint, percentage infarct size post‐PPCI (as measured by cardiac MRI) was compared at 12 weeks between groups using the 2‐sample *t* test. This analysis was then repeated using multiple linear regression with adjustment for all stratification variables used during the course of the trial (infarct location [anterior or nonanterior], sex and time from symptom onset to randomisation [0 to <3 h or 3 to ≤6 h]). In light of the achieved sample size, and the publication of the CONSORT extension guidelines for pilot trials,^17^, ^18^ the study has primarily been reported broadly in line with these, without formal hypothesis testing of outcome measures other than a 12 week between groups comparison of the primary outcome (percentage infarct size post‐PPCI as measured by cardiac MRI) using the 2‐sample *t* test. Outcomes were summarised by group while additional, exploratory modelling is presented in supplemental tables.

## 3 RESULTS

### 3.1 Patient population

**Figure 1:**
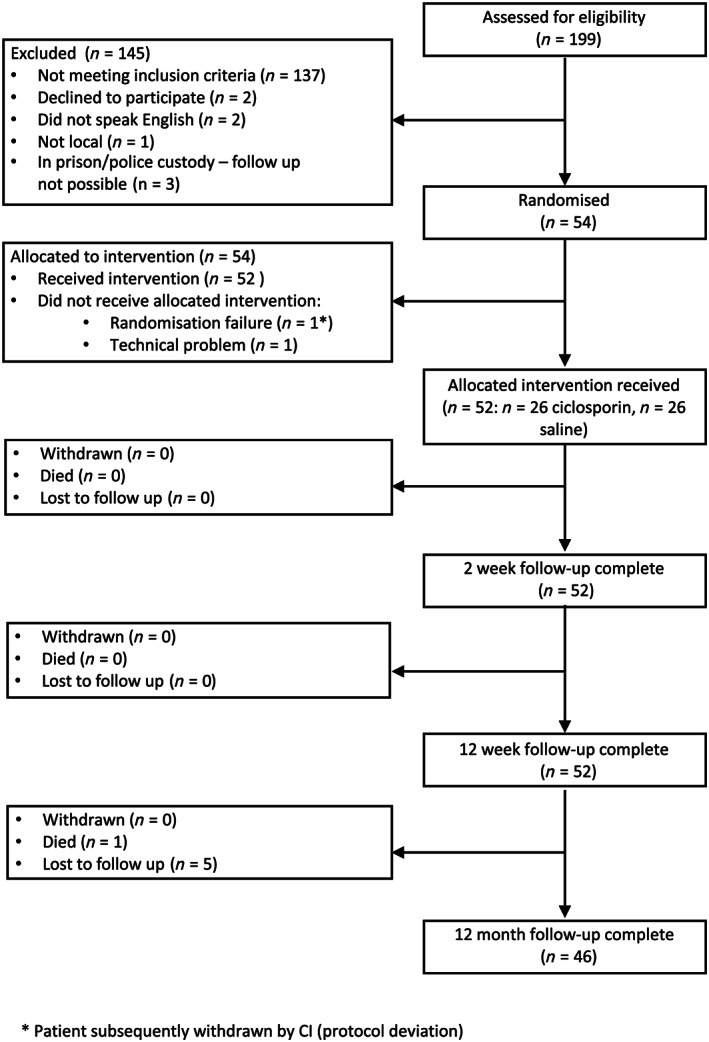
CONSORT diagram with participant flow for each group, losses and exclusions after randomisation

Out of 199 screened patients (Figure 1), 54 participants were recruited into the trial but only 52 were randomised to 1 of the 2 arms. One participant was not included in the trial due to a problem encountered with viewing the allocation arm from the randomisation website and the second was due to a technical problem with opening the prescription. Participants were predominantly male, past or nonsmokers with little previous serious medical history or ongoing diabetes (Table 1). Participants in the control arm were slightly older with the median age being 66 years (interquartile range: 60.7–74.2) compared to 61.3 years (interquartile range: 53.7–70.7) in ciclosporin arm. Over 2‐thirds of patients had their infarct in the non‐anterior location (69% in each arm). Generally, all baseline characteristics were reasonably well balanced between trial arms.

**Table 1:** Baseline characteristics preadmission by arm

	Control (*n* = 26)		Ciclosporin (*n* = 26)	
Categorical variables	*n*	(%)	*n*	(%)
**Sex:**				
**Male**	20	77	24	92
**Diabetes:**				
**Yes**	2	8	2	8
**Smoking status:**				
**Never**	9	35	9	34.5
**Past**	12	46	9	34.5
**Current**	5	19	8	31
**Medical history:**				
**Ischaemic heart disease**	0	0	3	11.5
**Previous myocardial infarction**	0	0	0	0
**CVA/TIA**	0	0	0	0
**COPD**	2	8	0	0
**Peripheral vascular disease**	1	4	0	0
**Previous PCI**	0	0	0	0
**Other relevant history**	6	23	11	42
**Medication pre‐admission:**				
**Aspirin**	4	15	1	4
**Clopidogrel/prasugrel/ticagrelor**	0	0	0	0
**Beta‐blocker**	1	4	2	8
**ACE inhibitor or angiotensin receptor blocker**	2	8	6	23
**Diuretic**	3	11.5	1	4
**Statin**	6	23	6	23
**Calcium channel blocker**	4	15	3	11.5
**Nitrates**	0	0	1	4
**Nicorandil**	0	0	1	4
**Infarct location:**				
**Non‐anterior**	18	69	18	69
**Anterior**	8	31	8	31

Continuous variables	*n*	Median (IQR)	Range	*n*	Median (IQR)	Range
**Age (y)**	26	66.0 (60.7–74.2)	50.5–83.8	26	61.3 (53.7–70.7)	43.7–91.5
**Body mass index**	26	28.1 (24.1–31.7)	19.1–37.9	26	28.7 (24–31)	22.1–37.3
**Time from symptom onset to randomisation (min)**	26	164 (120–227)	64–366	26	162 (94–272)	72–362

COPD, chronic obstructive pulmonary disease; CVA, cerebrovascular accident; IQR, interquartile range; PCI, percutaneous coronary intervention; TIA, transient ischaemic attack.

### 3.2 Peri‐interventional parameters

There were also no notable or apparent clinically important differences in peri‐interventional characteristics (Table 2) between arms. Time from symptom onset to reperfusion was around 3 hours in both arms, and door‐to‐balloon time remained under 30 minutes despite randomisation and trial drug infusion prior to reperfusion. Periprocedural antiplatelet therapy (ticagrelor, prasugrel) was similar in both arms, and TIMI 3 flow was achieved in 50 out of 52 patients. Target vessel diameter and stented length were similar in both arms. Discharge medication included dual antiplatelet therapy and atorvastatin in all patients, and almost all patients received a β‐blocker (50/52) and an angiotensin converting enzyme inhibitor (49/52; Table S1).

**Table 2:** Peri‐interventional characteristics by arm

	Control (*n* = 26)		Ciclosporin (*n* = 26)	
Variable	*n*	(%)	*n*	(%)
**Peri‐interventional medication:**				
**Aspirin**	26	100	26	100
**Clopidogrel**	1	4	4	15
**Prasugrel**	21	81	19	73
**Ticagrelor**	4	15	3	11.5
**Abciximab**	0	0	1	4
**Tirofiban**	15	58	19	73
**Bivalirudin**	0	0	0	0
**Heparin**	26	100	26	100
**Heparin units**				
**≤5000**	17	65.5	19	73
**>5000**	9	34.5	7	27
**PCI‐related parameters:**				
**Culprit lesion**				
**LAD**	7	27	8	31
**LCx**	4	15	7	27
**RCA**	15	58	11	42
**TIMI flow before**				
**0**	18	69	25	96
**1**	8	31	1	4
**2**	0	0	0	0
**3**	0	0	0	0
**TIMI flow post**				
**0**	0	0	0	0
**1**	2	8	0	0
**2**	0	0	0	0
**3**	24	92	26	100
**Aspiration catheter (y/*n*)**	7	27	6	23
**Reperfusion arrhythmias (y/*n*)**	10	38.5	13	50

Continuous variables	*n*	Median (IQR)	Range	*n*	Median (IQR)	Range
**PCI procedure:**						
**Onset to balloon (min)**	26	167.5 (107–241)	46–376	26	182 (116–287)	76–371
**Call to balloon (min)**	26	59.5 (50–79)	40–170	26	72 (51–90)	39–140
**Door to balloon (min)**	26	25.5 (23–33)	12–90	26	27 (22–33)	17–59
**Onset of symptoms to reperfusion**	26	175.5 (130–240)	69–380	26	189 (118–291)	72–371
**Target vessel diameter (mm)**	26	3.5 (3–3.7)	3–5	26	3.5 (3.5–4)	3–5
**Target vessel stent length (mm)**	26	33 (23–56)	0–89	26	37 (26–52)	7.5–114
**Troponin T at admission (0 h; ng/L)** ^*^	26	46.5 (30–73)	12–799	26	58 (27–96)	7–1026
**Troponin T at 12 h post PPCI (ng/L)** ^*^	26	3864 (1169–5659)	441–10 000	26	3414 (1190–6981)	333–10 000

* laboratory lists any troponin over 10000 as 10 000 exactly.

### 3.3 Ciclosporin pharmacokinetics and safety

**Figure 2:**
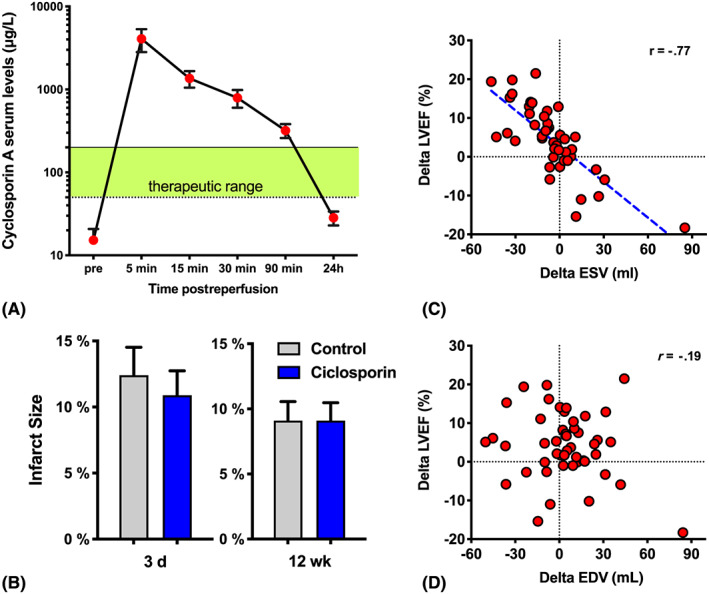
Ciclosporin does not affect cardiac remodelling post infarction. (A) Ciclosporin serum level kinetics for *n* = 8 randomly selected patients from ciclosporin group (displayed as mean ± standard error of the mean per time point). (B) Infarct size after 3 days and 12 weeks. (C, D) correlation of changes in left ventricular ejection fraction (LVEF), end‐diastolic volume (EDV) and end‐systolic volume (ESV) in 48 patients with both magnetic resonance images (Spearman r)

A full‐time course of ciclosporin serum levels was determined in 10 randomly selected patients from the intervention arm, of whom 8 had a complete time course. Postreperfusion serum levels (at 5 min) exceeded 4 mg/L, then quickly dropped to 0.2 mg/L at 90 minutes and 0.02 mg/L 24 hours later (Figure 2A). In the Piot *et al*. study, ciclosporin concentrations were 6272 ± 714 ng/mL after 1 minute, compared to ours (4068 ± 1254 ng/mL [4 mg/L]) after 5 minutes. We both saw a sufficient blood concentration for at least 90–180 minutes (therapeutic level 50–200 ng/mL). Following acute PCI, renal function deteriorated in both groups, indicated by a rise in creatinine on day 3 (23% increase in control group *vs* 18% in ciclosporin group, Table S1). However, there was no significant difference between groups. Adverse events (AEs) occurred in 18 patients in placebo arm (6 patients with ≥2 AEs), and 14 AEs in 11 patients in ciclosporin arm (3 patients with ≥2 AEs). Finally, 23 contrast‐induced nephropathy AEs were classified as *serious.*

### 3.4 Clinical endpoints

Few clinical events were observed in the trial at 12 months with just a solitary death (caused by intracranial bleeding) and a nonfatal case of stroke/cerebrovascular accident (chronic subdural haematoma) meeting the definition of a secondary outcome clinical event (death from any cause, stroke or myocardial infarction). Both clinical events occurred to participants in the ciclosporin arm after the end of the 12‐week randomised controlled trial but before the final study follow‐up at 12 months. Other clinical events were observed in 3 participants, which included angina and chest pain but these events were not deemed to meet the definition of secondary outcome clinical events in the trial).

### 3.5 Effect of ciclosporin on infarct size and LV remodelling

All 52 patients attended the 12‐week follow‐up visit for the cardiac MRI, but in 4 patients (all from the intervention arm) 1 of their 2 MRI scans was not analysable (technical problems with scanner or insufficient image quality, e.g. atrial fibrillation). The unadjusted mean infarct size at 12 weeks was identical in both arms (mean = 9.1, standard deviation [SD] = 7.0, Figure 2B), and when the distribution of covariates was taken into account, there was still little or no difference in mean percentage infarct size between arms (adjusted mean difference of −0.1 (95% confidence interval [CI] −2.4 to 2.2). While the 95% CIs are wide, they do not extend beyond the clinically important difference of 4.6% used to establish the sample size. The absolute reduction of 5% deemed to be of clinically important significance^19^ was also not observed. Finally, we looked at LV remodelling, comparing the 12‐week MRI scans to the baseline MRI scans (Table 3). There was a nonsignificant reduction in infarct size in both treatment arms (2.4 *vs* 1.8%), probably owing to discrete overestimation at baseline secondary to myocardial oedema.^20^, ^21^ LVEF appeared to improve in both arms at 12 weeks from baseline, and may have been greater improvement in control arm than intervention arm (6.8% (SD = 7.6) *vs* 2.1% (SD = 8.8), Table 3 and Table S4). While a change in EDV is important for remodelling, only changes in ESV appeared to correlate with ΔLVEF (*r* = −0.77, Figure 2C,D). Hence, LV volumes appeared to be more reduced in the control arm, especially difference for ESV (ΔESV −12.2 *vs* +1.4 mL; ΔEDV −2.0 *vs* +9.1 mL, Table S3).

**Table 3:** Cardiac magnetic resonance imaging and late gadolinium enhancement data at baseline and 12 weeks by arm

	Control						Ciclosporin					
	Baseline			12 weeks			Baseline			12 weeks		
Outcome	n	Median (IQR)	Range	n	Median (IQR)	Range	n	Median (IQR)	Range	n	Median (IQR)	Range
**Infarct size (%)**	25	10 (7.1–15.3)	0.9–54.7	23	9.1 (3–13.9)	0–28.2	26	9.5 (2.9–14.5)	0.2–36.8	26	9.8 (4.2–11.4)	0–27.2
**Myo vol (mL)**	25	123.5 (102.4–147.7)	79.6–225.6	23	105.8 (89.4–111.7)	63–145.2	26	133.5 (113.5–144)	74.8–182.9	26	111.5 (97–127.4)	54.2–146.6
**Myo mass (g)**	25	129.6 (107.5–155.1)	80.7–236.9	23	111.1 (93.9–117.3)	66.1–152.4	26	140.1 (119.2–151.2)	78.5–192.1	26	117.1 (101.9–133.8)	56.9–154
**Infarct vol (mL)**	25	11.8 (7.6–20.5)	0.9–66.5	23	8.2 (2.9–15.2)	0–30.1	26	12.0 (2.3–21.3)	0.2–54.8	26	10.0 (4.3–14.1)	0–38.2
**Infarct mass (g)**	25	12.3 (8–21.6)	0.9–69.8	23	8.6 (3–16)	0–31.6	26	12.6 (2.4–22.4)	0.2–57.6	26	10.4 (4.5–14.8)	0–40.1
**MVO mass (g)**	25	0.2 (0–1.6)	0–17.9		NA	NA	26	0 (0–0.4)	0–32.4		NA	NA
**EDV (mL)**	25	156.6 (137.7–173.8)	103.9–225.8	23	153.5 (136.1–171.5	92.6–240.7	26	161.9 (140.9–178.1)	115.8–195.5	26	173.9 (147.3–193.5)	103–238.8
**ESV (mL)**	25	80.9 (68–91.3)	40.7–152.2	23	67.8 (49.6–85.8)	26.6–120.4	26	75.4 (67.8–97)	38–113.2	26	75.6 (60.3–95.4)	35.5–160.9
**SV (mL)**	25	76.6 (63.2–85.8)	31.9–105.8	23	84.3 (72.4–94.7)	50.6–121.1	26	80.2 (69.8–89.2)	59.5–111.7	26	82.6 (74.7–103.5)	54–119.9
**LVEF (%)**	25	49.2 (38.5–56.5)	25.6–66.8	23	55.1 (48.7–64.2)	35.5–71.2	26	53.5 (44.1–56.9)	36.5–67.8	26	53.2 (49–60.7)	32.6–65.5
**Myo mass diastole (g)**	25	107.8 (86.5–118.5)	67.5–203.5	23	91.8 (81.1–105.2)	58.1–134.5	26	113.9 (105.7–135.2)	76.2–162.6	26	101.2 (81.9–114.5)	61.3–132.5
**Myo mass systole (g)**	25	113.1 (92.5–121.1)	68.8–201.3	23	98.7 (85.4–107.5)	59.9–137.5	26	119.9 (109.6–136.7)	78.8–180.8	26	108.4 (85.4–125.8)	55.6–156.7

### 3.6 Lymphocyte kinetics in myocardial ischaemia/reperfusion

To elucidate the impact of a single ciclosporin bolus on a proximal readout, each continuous secondary outcome was compared at baseline between arms or at short snapshots in time as appropriate (0–90 min, 24 h). A statistically significant change in T lymphocyte counts (CD3) between arms was only observed at 5 minutes relative to baseline but this effect was no longer present from 15 minutes onwards (Tables S4a‐g). The unadjusted mean CD3 counts were not significantly higher in the ciclosporin arm at 5 minutes compared to the control arm, but when the distribution of covariates were taken into account they were significantly higher in the ciclosporin arm (adjusted mean difference of 142.4 [95% CI: 23.9 to 260.9] Tables S5 and S6). The mean drop (relative to prereperfusion) of CD3 T‐lymphocytes at 90 minutes was 26% in the control arm *vs* 37% in the ciclosporin arm. At 24 hours (Table S6) and 2 weeks postreperfusion, there was no statistical difference between control and ciclosporin arm for CD4 T‐cells, CD8 T‐cells, natural killer cells, and B‐lymphocytes, suggesting no antiproliferative effect of ciclosporin bolus on any lymphocyte population.

## 4 DISCUSSION

The results of the CAPRI pilot trial, as did the recently reported CYCLE and CIRCUS multicentre trials, add further evidence to the lack of effect of ciclosporin on myocardial ischaemia/reperfusion injury in patients with acute STEMI. This suggests that mitochondrial pore inhibition by ciclosporin or similar drugs does either not reach ischaemic cardiomyocytes or requires earlier application. Alternatively, it is possible that in this era with stent‐based revascularisation under optimal antiplatelet therapy myocardial ischaemia/reperfusion injury is clinically less relevant than previously thought.^11^ Almost all of the CAPRI patients received either ticagrelor or prasugrel in addition to aspirin prior to reperfusion. These results are a further indication that translation of successful animal studies into the clinic is far from straight forward, probably because of the nature of experimental models. None of these studies so far has been able to model plaque rupture with thrombotic load and a comparable response of the adaptive immune system.

While previous trials have considered some of the methodological issues, we have addressed the following matters: (i) a fully blinded (and not open‐label), randomised design; (ii) confirmed optimal drug concentration and pharmacokinetics; (iii) used cardiac MRI imaging with gadolinium contrast as a gold‐standard rather than echocardiography to quantify acute infarct size; (iv) performed a second MRI at 3 months to quantify remodelling as well as late infarct size; and finally (v) used polychromatic flow cytometry to meticulously document the time course of all major lymphocyte subsets during reperfusion and 2‐week recovery, given that CX3CR1‐dependent T‐lymphocyte marginalisation coincides with microvascular obstruction and determines long term mortality in these patients.^16^

### 4.1 LV remodelling post‐STEMI

Despite not finding evidence of a statistical or clinically meaningful difference in the primary outcome, the design of the CAPRI trial enabled us to look at additional parameters in order to capture further effects of ciclosporin, if existent. CAPRI is the largest MRI ciclosporin trial to quantify infarct size as well as LV remodelling. To our surprise, despite identical scar size at 12 weeks post‐MI, remodelling in the control trial arm appeared more favourable than in the ciclosporin arm. This appeared to be driven by the higher number of TIMI 1 patients prereperfusion. A reduction in ESV, but not EDV, appeared to be the main determinant of improved LV function after 12 weeks. Unlike CAPRI, CYCLE was an open label trial and CIRCUS used a different formulation of ciclosporin. In addition, neither of the 2 previous studies used cardiac MRI to assess their endpoints. Two‐dimensional echocardiography without the use of a contrast dye is not able to quantify infarct size, and it is the least accurate method to determine LV volumes. The raw data of the CYCLE and CIRCUS trial also show that LV volumes in a similar patient population differ by almost 100% between these 2 studies, possibly due to different formulas to estimate LV volumes. The CIRCUS trial has looked at remodelling purely based on 2‐dimensional echocardiography without ultrasound contrast (Simpson formula). Measurements of LVEF by 2‐dimensional echocardiography have been tarnished by significant observer variability and poor agreement with reference methods.^22^, ^23^, ^24^ LV opacification by intravenous ultrasound contrast agents improves both.^25^

### 4.2 T‐cell response and ciclosporin in STEMI

The role of T‐lymphocytes in infarct healing post STEMI has not been investigated before in clinical trials^26^, ^27^; however, Weirather and colleagues have previously demonstrated the role of regulatory T‐cells in the remodelling process in a mouse model.^28^ The Frangogiannis group has also identified in the mouse that CCR5 signalling suppresses inflammation and reduces adverse remodelling of the infarcted heart, mediating recruitment of regulatory T cells.^29^ We have previously shown that the magnitude of the proinflammatory Th1 response from CD4 T‐cells in acute STEMI patients correlates with total ischaemic time.^30^ We also identified a proliferative response among CD4 T‐cells during the first 24 hours postreperfusion,^31^ which has now been confirmed in the CAPRI trial (Online Table 4a‐g). While in vitro experiments demonstrate that differentiation from naïve into Th1 cells is inhibited by ciclosporin,^32^^,^ 33 the CAPRI trial did not identify an antiproliferative effect of ciclosporin during the first 24 hours, despite pharmacologically relevant serum levels during the first hours following bolus application.

### 4.3 Alternative mechanism and timing for ciclosporin therapy in STEMI

One of the reasons why ciclosporin did not demonstrate additional protection could have been the co‐medication with ticagrelor,^34^, ^35^, ^36^ prasugrel or ACE inhibitors,^37^, ^38^ which have all demonstrated favourable effects in patient outcome by themselves. Many of our patients have call to balloon times of over an hour despite short door to balloon times. Especially when patients are admitted to an emergency room in a noninterventional hospital, interhospital transfer adds a significant delay to successful reperfusion. In this trial all patients had ciclosporin been given immediately (5 min) before successful reperfusion. It is possible that ciclosporin is not so effective for *reperfusion injury* (which is not a proven phenomenon in human patients), but rather in ischaemic cardiomyocytes and endothelial cells, preventing or delaying ongoing apoptosis in those cell types. Ciclosporin (10 μmol/L) completely inhibited the oxLDL‐induced release of cytochrome C in a study by Walter and colleagues.^39^ Moreover, tumour necrosis factor‐α‐ and angiotensin II‐induced cytochrome C release was also prevented by ciclosporin treatment. There is a clinical window of at least 60 minutes in most STEMI patients where, other than pain medication and aspirin, basically no therapy is present. Application of ciclosporin 60 minutes before reperfusion could have a significant effect on infarct size, if it prevents cardiomyocyte or endothelial cell apoptosis in vivo at the given concentration.

## 5 CONCLUSIONS

We confirm in this clinical trial that intravenous administration of the mitochondrial pore inhibitor ciclosporin before reperfusion by primary coronary angioplasty is safe, but does not affect myocardial injury. Serum levels of ciclosporin during 90 minutes of reperfusion were 10‐fold higher than established therapeutic levels for immunosuppression, suggesting that either (i) ciclosporin does not reach ischaemic cardiomyocytes, or (ii) ciclosporin needs to be given at an earlier timepoint during myocardial ischaemia (e.g. before transfer for primary PCI). Also, 1 bolus of ciclosporin is not sufficient to inhibit CD4 T‐lymphocyte proliferation during remodelling. Given that ciclosporin is safe in STEMI patients, we suggest conducting a new randomised controlled trial where ciclosporin is given at the first point of contact (e.g. ambulance) and also test prolonged therapy in order to inhibit T‐cell proliferation during remodelling.

## ACKNOWLEDGEMENTS

The research was funded/supported by grants to I.S. by the National Institute for Health Research Newcastle Biomedical Research Centre based at Newcastle upon Tyne Hospitals NHS Foundation Trust and Newcastle University, and the British Heart Foundation (PG/18/25/33587). The views expressed are those of the author(s) and not necessarily those of the NHS, the National Institute for Health Research or the Department of Health.

## COMPETING INTERESTS

There are no competing interests to declare.

## CONTRIBUTORS

Grant application and study design: I.S. Statistical/Data analysis: T.C. and A.B. Trial manager: A.S. Manuscript writing: I.S., A.S., T.C., A.B., S.C., M.E., K.S. Cardiac MRI analysis: R.D. and A.M. Flow cytometry analysis: P.P. and S.C.

## DATA AVAILABILITY STATEMENT

Anonymised data from this trial may be available to the scientific community subject to regulatory and ethics approval. Requests for anonymised data should be directed to the corresponding author. The authors confirm that the Principal Investigator for this paper is I.S. and that he/had direct clinical responsibility for patients.

## Supporting information


**Table S1:** Discharge medication by arm
**Table S2**: Renal function outcomes by arm
**Table S3**: Change in late gadolinium enhancement and cardiac magnetic resonance imaging data at 12 weeks from baseline by arm
**Table S4a**: Lymphocyte counts and sub‐cell types at baseline (0 min) by arm
**Table S4b**: Lymphocyte counts and sub‐cell types at 5 minutes by arm
**Table S4c**: Lymphocyte counts and sub‐cell types at 15 minutes by arm
**Table S4d**: Lymphocyte counts and sub‐cell types at 30 minutes by arm
**Table S4e**: Lymphocyte counts and sub‐cell types at 90 minutes by arm
**Table S4f**: Lymphocyte counts and sub‐cell types at 24 hours by arm
**Table S4g**: Lymphocyte counts and sub‐cell types at 14 days by arm
**Table S5**: Multiple linear regression showing continuous secondary outcomes between arms
**Table S6**: Multiple linear regression showing post‐hoc continuous secondary outcomes between arms at 24 hours

Data S1. Supporting Info Item
