# Supplementary material for: Effect of ciclosporin on safety, lymphocyte kinetics and left ventricular remodelling in acute myocardial infarction
Source: Br J Clin Pharmacol. 2020 Mar 11;86(7):1387–97. doi: 10.1111/bcp.14252 (PMC7318996; doi:10.1111/bcp.14252)
Supplement: Supplementary file 1 — Table S1: Discharge medication by arm Table S2: Renal function outcomes by arm Table S3: Change in late gadolinium enhancement and cardiac magnetic resonance imaging data at 12 weeks from baseline by arm Table S4a: Lymphocyte counts and sub‐cell types at baseline (0 min) by arm Table S4b: Lymphocyte counts and sub‐cell types at 5 minutes by arm Table S4c: Lymphocyte counts and sub‐cell types at 15 minutes by arm Table S4d: Lymphocyte counts and sub‐cell types at 30 minutes by arm Table S4e: Lymphocyte counts and sub‐cell types at 90 minutes by arm Table S4f: Lymphocyte counts and sub‐cell types at 24 hours by arm Table S4g: Lymphocyte counts and sub‐cell types at 14 days by arm Table S5: Multiple linear regression showing continuous secondary outcomes between arms Table S6: Multiple linear regression showing post‐hoc continuous secondary outcomes between arms at 24 hours [file BCP-86-1387-s001.doc]

**Suppl. Table 1: Discharge medication by arm**

|  | **Control (n=26 )** | | **Ciclosporin (n=26 )** | |
| --- | --- | --- | --- | --- |
| **Categorical variables** | **n** | **(%)** | **n** | **(%)** |
| **Medication at discharge**  **Aspirin**  **Clopidogrel**  **Prasugrel**  **Ticagrelor**  **Betablocker**  **ACEi or ARB**  **Diuretic**  **Statin**  **Ca Blocker**  **Nitrates**  **Nicorandil** | 26  1  17  8  26  23  2  26  1  2  1 | 100  4  65  31  100  88  8  100  4  8  4 | 26  1  20  5  24  26  4  26  0  1  1 | 100  4  77  19  92  100  15  100  0  4  4 |

**Suppl. Table 2: Renal function outcomes by arm**

|  | **Control (n=26 )** | | | | **Ciclosporin (n=26 )** | | | |
| --- | --- | --- | --- | --- | --- | --- | --- | --- |
| **Continuous variables** | **n** | **Mean**  **(SD)** | **Median**  **(IQR)** | **Range** | **n** | **Mean**  **(SD)** | **Median**  **(IQR)** | **Range** |
| **Renal function at baseline:** | | | | | | | | |
| **Glomerular filtration rate (GFR) at baseline** | 26 | 87  (25.1) | 82.5  (70-93) | 34-163 | 26 | 87.3  (24.9) | 83  (74-93) | 51-147 |
| **Contrast volume per GFR (ml)** | 26 | 132.9  (51.9) | 120  (90-160) | 75-250 | 26 | 154.2  (58.6) | 160  (110-180) | 60-300 |
| **Urea at (days):**  **0**  **1**  **2**  **3**  **14** | 26  26  20  25  26 | 5.9  (2.9)  5.2  (2.2)  5.0  (1.4)  6.6  (2.6)  5.5  (1.7) | 5.4  (4.7-7.3)  5.1 (3.6-6.3)  4.8  (4-6.6)  6.4  (5.1-8.4)  5.1  (4.4-6.5) | 2.4-17.3  2.6-13.2  2.3-7.3  1.8-14.1  3-9.7 | 26  25  20  25  26 | 5.7  (1.4)  5.1  (1.9)  5.2  (1.9)  6.3  (2.2)  5.5  (1.3) | 5.8 (4.5-6.7)  4.7 (3.9-6.6)  4.6  (3.6-5.8)  5.7  (4.9-6.6)  5.5  (4.6-6.2) | 2.7-8.6  2-10.6  3.1-10.2  3.6-12  3.2-8.4 |
| **Creatinine at (days):**  **0**  **1**  **2**  **3**  **14** | 26  26  20  25  26 | 83.7  (25.6)  92.1  (22.4)  93.4  (27.0)  103.3  (28.9)  94.5  (22.0) | 81  (73-97)  92  (79-105)  91  (78-96)  98  (91-116)  96.5  (81-107) | 37-178  48-158  48-182  43-173  41-146 | 26  25  20  25  26 | 85.2  (17.7)  93.8  (19.2)  96.9  (20.7)  100.4  (21.4)  93.5  (18.3) | 88  (72-94)  94  (82-106)  96.5  (85.5-109.5)  100  (89-111)  94  (81-105) | 44-125  54-132  56-137  62-158  55-144 |

| **Suppl. Table 3: Change in LGE and cardiac MRI data at 12 weeks from baseline by arm** | | | | | | | | |
| --- | --- | --- | --- | --- | --- | --- | --- | --- |
|  | **Control** | | | | **Ciclosporin** | | | |
| **Outcome** | **n** | **Mean (SD)** | **Median**  **(IQR)** | **Range** | **n** | **Mean (SD)** | **Median**  **(IQR)** | **Range** |
| **LGE data** | | | | | | | | |
| **Infarct size (%) (Primary outcome)** | 22 | -2.4  (6.6) | -1.2  (-4.8 to 0.7) | -26.5 to 6.6 | 26 | -1.8  (4.8) | -2.6  (-5.1 to 1.5) | -10.1 to 7.9 |
| **Myo vol (ml)** | 22 | 25.2  (21.1) | -23.3  (-41.9 to -14.1) | -80.4 to 13.9 | 26 | -18.9  (17.6) | -17.9  (-30.2 to -8.2) | -58.4 to 11.0 |
| **Myo mass (g)** | 22 | -26.6  (22.2) | -24.5  (-44.0 to -14.8) | -84.5 to 14.6 | 26 | -19.8  (18.4) | -18.8  (-31.7 to -8.6) | -61.4 to 11.5 |
| **Infarct vol (ml)** | 22 | -5.7 (8.9) | -3.8  (-8.2 to -1.2) | -36.4 to 5.8 | 26 | -4.5  (7.5) | -2.6  (-8 to 1.2) | -25.4 to 6.7 |
| **Infarct mass (g)** | 22 | -6.0  (9.4) | -4.0  (-8.6 to -1.3) | -38.2 to 6.2 | 26 | -4.8  (7.9) | -2.7  (-8.4 to 1.2) | -26.7 to 7.1 |
| **Cardiac MRI data** | | | | | | | | |
| **EDV (ml)** | 22 | -2.0  (19.4) | 2.5  (-10 to 9.6) | -50.3 to 31.7 | 26 | 9.1 (27.2) | 8.6  (-2 to 23.9) | -45.3 to 84.2 |
| **ESV (ml)** | 22 | -12.2 (16.0) | -9.4  (-20.4 to -0.5) | -46.4 to 14.6 | 26 | 1.4 (23.7) | -2  (-9 to 7.7) | -35.5 to 85.1 |
| **SV (ml)** | 22 | 10.2 (14.9) | 14.7  (-0.3 to 22) | -20.7 to 32.3 | 26 | 7.7 (17.3) | 7.4  (-0.9 to 14.3) | -29.8 to 60.4 |
| **LVEF (%)** | 22 | 6.8 (7.6) | 7.3  (1.7 to 12.9) | -11 to 19.8 | 26 | 2.1 (8.8) | 2.5  (-1 to 6.1) | -18.3 to 21.5 |
| **Myo mass**  **diastole (g)** | 22 | -18.0 (19.7) | -13.4  (-29.6 to -2.7) | -69 to 10 | 26 | -18.9 (17.0) | -19.5  (-29.3 to -8.9) | -57.5 to 27 |
| **Myo mass**  **systole (g)** | 22 | -14.1 (15.1) | -13.0  (-21.5 to -2.8) | -63.8 to 4 | 26 | -17.3 (22.6) | -13.8  (-24.8 to -5.4) | -69.5 to 33.4 |

**Suppl. Table 4a: Lymphocyte counts and sub-cell types at baseline (0 mins) by arm**

|  | **Baseline (0 mins)** | | | | | | | |
| --- | --- | --- | --- | --- | --- | --- | --- | --- |
|  | **Control** | | | | **Ciclosporin** | | | |
| **Outcome** | **n** | **Mean (SD)** | **Median (IQR)** | **Range** | **n** | **Mean (SD)** | **Median (IQR)** | **Range** |
| **Lymphocytes (total) (CD45)** | 26 | 1842  (935) | 1567  (1096- 2531) | 924-4454 | 26 | 2068  (691) | 1958  (1560- 2683) | 862-3445 |
| **B-cells (CD19)** | 26 | 239  (332) | 146  (121-230) | 36-1780 | 26 | 201  (107) | 166  (127-294) | 38-424 |
| **NK-cells** | 26 | 423  (275) | 367  (239-527) | 69-1140 | 26 | 500  (295) | 414  (301-674) | 73-1418 |
| **T-cells (CD3)** | 26 | 1169  (688) | 978  (706-1470) | 437-3696 | 26 | 1350  (540) | 1277  (997-1622) | 372-2329 |
| **CD4** | 26 | 785  (532) | 624  (461-861) | 256-2905 | 26 | 785  (294) | 771  (585-935) | 300-1489 |
| **CD4 Naive** | 26 | 363  (362) | 268  (223- 388) | 60-1935 | 26 | 354  (199) | 303  (193- 504) | 56-790 |
| **CD4 CM** | 26 | 348  (191) | 332  (249- 421) | 12-921 | 26 | 343  (142) | 339  (239- 411) | 110-639 |
| **CD4 EM** | 26 | 58.8  (56.3) | 44.8  (23- 61.4) | 0.7-254.1 | 26 | 66.6  (72.7) | 48.5  (20.4- 77.5) | 1-307.7 |
| **CD4 TEMRA** | 26 | 15.6  (22.2) | 3.8  (1.4- 19.4) | 0.8-74 | 26 | 22.1  (28.7) | 6.7  (1.7- 39.2) | 0.3-85.3 |
| **% CD69+CD4+** | 20 | 15.2  (12.8) | 12.3  (10.3- 14.7) | 6.9-67.7 | 21 | 14.3  (4.3) | 13.7  (10.6- 15.7) | 7.6-24.6 |
| **CD8** | 26 | 357  (267) | 250  (187- 431) | 18-994 | 26 | 504  (392) | 386  (280- 625) | 71-1667 |
| **CD8 Naive** | 26 | 89.9  (86.4) | 78.2  (33.6- 93.4) | 7.6-400.8 | 26 | 107.9  (74.6) | 87.1  (58.2- 149.2) | 22.1-357.7 |
| **CD8 CM** | 26 | 46.6  (27.1) | 44.8  (25- 62.4) | 0.9-99.8 | 26 | 57.9  (34.6) | 46.0  (33.2- 84.1) | 11.4-132.8 |
| **CD8 EM** | 26 | 42.1  (36.9) | 33.1  (24.5- 42.3) | 0-155.1 | 26 | 92.5  (162) | 29.2  (16.2- 62.4) | 4.3-757.4 |
| **CD8 TEMRA** | 26 | 178.9  (201) | 94.8  (72.5- 151.1) | 1.4-721.8 | 26 | 246.0  (249) | 190.9  (94.8- 280.4) | 24-1199 |
| **% CD69+CD8+** | 20 | 29.7  (17.3) | 25.0  (20.0- 32.4) | 10.2-91.1 | 21 | 26.1  (7.1) | 24.9  (20.7- 30.1) | 14.9-43 |

**Suppl. Table 4b: Lymphocyte counts and sub-cell types at 5 mins by arm**

|  | **At 5 mins** | | | | | | | |
| --- | --- | --- | --- | --- | --- | --- | --- | --- |
|  | **Control** | | | | **Ciclosporin** | | | |
| **Outcome** | **n** | **Mean (SD)** | **Median (IQR)** | **Range** | **n** | **Mean (SD)** | **Median (IQR)** | **Range** |
| **Lymphocytes (total) (CD45)** | 26 | 1497  (660) | 1399  (983- 1878) | 837-3597 | 26 | 2007  (734) | 1908  (1492- 2374) | 713-3518 |
| **B-cells (CD19)** | 26 | 196  (231) | 149  (105- 214) | 41-1266 | 26 | 205  (109) | 179  (137- 291) | 37-415 |
| **NK-cells** | 26 | 329  (198) | 300  (199- 398) | 54-867 | 26 | 542  (318) | 561  (233- 693) | 47-1231 |
| **T-cells (CD3)** | 26 | 963  (534) | 896  (662- 1146) | 366-3025 | 26 | 1248  (565) | 1146  (862- 1519) | 377-2532 |
| **CD4** | 26 | 656  (415) | 547  (431- 708) | 208-2384 | 26 | 719  (308) | 690  (550- 875) | 252-1696 |
| **CD4 Naive** | 26 | 309  (310) | 234  (168- 357) | 45-1690 | 26 | 314  (198 | 248  (174- 447) | 58-794 |
| **CD4 CM** | 26 | 290  (140) | 273  (211- 392) | 12-651 | 26 | 329  (153) | 303  (216- 412) | 101-750 |
| **CD4 EM** | 26 | 47.0  (48.1) | 34.9  (17.9- 54.2) | 0.9-321.2 | 26 | 59.0  (61.2) | 48.2  (22.3- 73.4) | 0.8-268.9 |
| **CD4 TEMRA** | 26 | 10.2  (13.2) | 3.1  (1.2- 18.6) | 0.5-47.5 | 26 | 17.0  (24.2) | 6.1  (1.9- 24.8) | 0-78 |
| **CD8** | 26 | 285  (212) | 209  (154- 313) | 16-806 | 26 | 471  (406) | 332  (239- 496) | 86-1849 |
| **CD8 Naive** | 26 | 76.1  (73.8) | 63.5  (31.8- 85.7) | 6.5-362.7 | 26 | 94.6  (76.9) | 74.8  (37.8- 130.3) | 15.2-384.4 |
| **CD8 CM** | 26 | 38.8  (25.0) | 33.8  (21.5- 55) | 1.6-90.2 | 26 | 53.7  (30.9) | 45.3  (30- 75.8) | 12.4-111.7 |
| **CD8 EM** | 26 | 34.2  (31.0) | 26.1  (16.8- 38.7) | 0.8-130.7 | 26 | 93.0  (168) | 34.5  (22.7- 57.7) | 3.2-810.1 |
| **CD8 TEMRA** | 26 | 136.3  (155) | 78.1  (57.4- 120) | 1-607.4 | 26 | 229.3  (265) | 156.4  (58.7- 207.2) | 34.1-1320 |

**Suppl. Table 4c: Lymphocyte counts and sub-cell types at 15 mins by arm**

|  | **At 15 mins** | | | | | | | |
| --- | --- | --- | --- | --- | --- | --- | --- | --- |
|  | **Control** | | | | **Ciclosporin** | | | |
| **Outcome** | **n** | **Mean (SD)** | **Median (IQR)** | **Range** | **n** | **Mean (SD)** | **Median (IQR)** | **Range** |
| **Lymphocytes (total) (CD45)** | 26 | 1363  (625) | 1160  (947- 1609) | 709-3502 | 26 | 1923  (885) | 1694  (1417- 2230) | 772-5084 |
| **B-cells (CD19)** | 26 | 195  (220) | 158  (102- 214) | 37-1181 | 26 | 197  (109) | 175  (113- 278) | 36-440 |
| **NK-cells** | 26 | 273  (154) | 252  (152- 355) | 57-607 | 26 | 525  (289) | 539  (284- 729) | 28-1035 |
| **T-cells (CD3)** | 26 | 888  (522) | 783  (583- 950) | 326-2959 | 26 | 1190  (685) | 1093  (830- 1252) | 360-3750 |
| **CD4** | 26 | 611  (413) | 509  (422- 684) | 199-2385 | 26 | 689  (418) | 622  (478- 773) | 241-2418 |
| **CD4 Naive** | 24 | 295  (319) | 234  (152- 331) | 45-1684 | 26 | 309  (234) | 236  (171- 393) | 41-1153 |
| **CD4 CM** | 24 | 276  (139) | 272  (191- 335) | 9-668 | 26 | 306  (184) | 269  (189- 362) | 97-1032 |
| **CD4 EM** | 24 | 43.3  (48.0) | 26.8  (16.0- 48.4) | 0.6-227.3 | 26 | 55.1  (53.3) | 47.9  (20.5- 66.2) | 0.8-244.6 |
| **CD4 TEMRA** | 24 | 8.1  (10.5) | 2.6  (0.8- 14) | 0.4-30.8 | 26 | 20.0  (31.3) | 4.1  (1.3- 26.9) | 0-120.9 |
| **CD8** | 26 | 258  (206) | 171  (140- 310) | 20-822 | 26 | 449  (390) | 306  (225- 470) | 91-1651 |
| **CD8 Naive** | 24 | 74.0  (79.8) | 55.0  (29.9-80.7) | 7.5-334.5 | 26 | 91.6  (111) | 68  (37.3-104.5) | 15.5-604.4 |
| **CD8 CM** | 24 | 37.2  (25.3) | 32.2  (19.8-54.2) | 0.8-96.7 | 26 | 48.7  (31.2) | 38.8  (22.2-68.4) | 12.8-136.5 |
| **CD8 EM** | 24 | 31.8  (32.5) | 22.4  (11.2-38) | 0-122.7 | 26 | 85.1  (160) | 31.6  (19.3-49.1) | 3.3-783.7 |
| **CD8 TEMRA** | 24 | 106.3  (133) | 58.5  (34.6-97.8) | 1-559 | 26 | 223.6  (243) | 158.0  (84.8-256.9) | 23-1186 |

**Suppl. Table 4d: Lymphocyte counts and sub-cell types at 30 mins by arm**

|  | **At 30 mins** | | | | | | | |
| --- | --- | --- | --- | --- | --- | --- | --- | --- |
|  | **Control** | | | | **Ciclosporin** | | | |
| **Outcome** | **n** | **Mean (SD)** | **Median (IQR)** | **Range** | **n** | **Mean (SD)** | **Median (IQR)** | **Range** |
| **Lymphocytes (total) (CD45)** | 26 | 1381  (798) | 1113  (977-1490) | 629-4342 | 26 | 1609  (675) | 1412  (1196-1919) | 557-3668 |
| **B-cells (CD19)** | 26 | 218  (305) | 153  (103-202) | 33-1627) | 26 | 209  (117) | 187  (119-317) | 38-490 |
| **NK-cells** | 26 | 266  (145) | 263  (179-331) | 40-650 | 26 | 386  (232) | 369  (197-523) | 18-958 |
| **T-cells (CD3)** | 26 | 886  (632) | 740  (558-928) | 305-3639 | 26 | 1006  (506) | 879  (719-1178) | 337-2694 |
| **CD4** | 26 | 638  (518) | 513  (406-696) | 191-2940 | 26 | 615  (316) | 558  (447-721) | 213-1808 |
| **CD4 Naive** | 24 | 325  (396) | 233  (166-354) | 42-2052 | 26 | 283  (189) | 223  (154-395) | 40-919 |
| **CD4 CM** | 24 | 281  (158) | 284  (207-328) | 12-835 | 26 | 270  (139) | 243  (188-334) | 87-718 |
| **CD4 EM** | 24 | 38.8  (39.9) | 19.7  (17.1-47.3) | 0.5-191.3 | 26 | 46.1  (47.4) | 35  (19.6-52.3) | 0.6-231.4 |
| **CD4 TEMRA** | 24 | 7.9  (9.8) | 2  (1-16.5) | 0.4-34.2 | 26 | 15.2  (23.8) | 3.6  (1.1-17.4) | 0.3-94 |
| **CD8** | 26 | 233  (166) | 179  (117-278) | 22-622 | 26 | 350  (282) | 253  (160-430) | 62-1119 |
| **CD8 Naive** | 24 | 74.6  (85.7) | 56.1  (21.7-86.3) | 7.6-414.5 | 26 | 75.2  (75.8) | 53.8  (28.1-106.4) | 13.5-398.8 |
| **CD8 CM** | 24 | 35.5  (25.1) | 31.2  (18.6-49.5) | 1.2-113.9 | 26 | 39.5  (24.7) | 32.6  (18.2-59.3) | 10.7-88.2 |
| **CD8 EM** | 24 | 27.1  (26.5) | 18.6  (10.8-38.2) | 0-120.8 | 26 | 67.2  (130) | 21.9  (13.8-40.7) | 2.3-641 |
| **CD8 TEMRA** | 24 | 95  (101) | 75.5  (31.5-92.7) | 2-365 | 26 | 168  (176) | 119.6  (52-188.4) | 10-792 |

**Suppl. Table 4e: Lymphocyte counts and sub-cell types at 90 mins by arm**

|  | **At 90 mins** | | | | | | | |
| --- | --- | --- | --- | --- | --- | --- | --- | --- |
|  | **Control** | | | | **Ciclosporin** | | | |
| **Outcome** | **n** | **Mean (SD)** | **Median (IQR)** | **Range** | **n** | **Mean (SD)** | **Median (IQR)** | **Range** |
| **Lymphocytes (total) (CD45)** | 26 | 1331  (742) | 1090  (824-1668) | 621-3942 | 26 | 1302  (596) | 1193  (963-1477) | 497-2943 |
| **B-cells (CD19)** | 26 | 210  (266) | 145  (100-214) | 36-1436 | 26 | 202  (113) | 168  (135-261) | 27-422 |
| **NK-cells** | 26 | 244  (188) | 231  (120-276) | 41-978 | 26 | 242  (171) | 203  (131-309) | 16-829 |
| **T-cells (CD3)** | 26 | 867  (572) | 757  (578-942) | 242-3290 | 26 | 852  (472) | 737  (504-1029) | 314-2313 |
| **CD4** | 26 | 657  (473) | 535  (444-769) | 164-2695 | 26 | 591  (326) | 585  (375-705) | 143-1617 |
| **CD4 Naive** | 26 | 333  (356) | 273  (185-315) | 35-1938 | 26 | 281  (195) | 245  (140-380) | 38-869 |
| **CD4 CM** | 26 | 283  (152) | 266  (179-364) | 14-722 | 26 | 274  (149) | 229  (173-367) | 61-650 |
| **CD4 EM** | 26 | 34.7  (31.1) | 24.9  (12.9-43.4) | 1-125.3 | 26 | 29.4  (20.4) | 23.6  (17.3-40.4) | 0.6-91.9 |
| **CD4 TEMRA** | 26 | 6.5  (8.8) | 2.4  (0.8-9.6) | 0.4-41.3 | 26 | 6.7  (9.5) | 2.2  (0.9-10.1) | 0-43.7 |
| **% CD69+CD4+** | 17 | 13.8  (10.9) | 10.4  (8.4-13.8) | 4-52.7 | 18 | 12.1  (3.4) | 11.8  (10-13.5) | 7-19.5 |
| **CD8** | 26 | 199  (137) | 148  (94-281) | 18-539 | 26 | 241  (183) | 150  (122-293) | 47-713 |
| **CD8 Naive** | 26 | 69.8  (74.8) | 54.1  (22.9-91) | 7.2-380.8 | 26 | 81.5  (97.4) | 46.1  (23.9-93.8) | 10.8-398.9 |
| **CD8 CM** | 26 | 33.2  (28.1) | 27.6  (13.4-44.9) | 0.7-141.6 | 26 | 40.6  (48.9) | 19.5  (13.6-61.6) | 12.4-240.2 |
| **CD8 EM** | 26 | 21.2  (21.3) | 12.6  (8.6-33.3) | 0.3-83.7 | 26 | 33.4  (49.6) | 17.0  (7.7-33.7) | 3.2-238.1 |
| **CD8 TEMRA** | 26 | 74.6  (64.6) | 56.2  (20.5-109.9) | 0.8-211.4 | 26 | 85.4  (78.4) | 58.4  (35.1-116.8) | 14.7-377.3 |
| **% CD69+CD8+** | 17 | 25.5  (18.8) | 19.6  (15.4-28.6) | 11.3-91.5 | 18 | 20.4  (5.5) | 20.6  (17.2-24.6) | 9.7-27.9 |

**Suppl. Table 4f: Lymphocyte counts and sub-cell types at 24 hours by arm**

|  | **At 24 hours** | | | | | | | |
| --- | --- | --- | --- | --- | --- | --- | --- | --- |
|  | **Control** | | | | **Ciclosporin** | | | |
| **Outcome** | **n** | **Mean (SD)** | **Median (IQR)** | **Range** | **n** | **Mean (SD)** | **Median (IQR)** | **Range** |
| **Lymphocytes (total) (CD45)** | 26 | 1997  (971) | 1687  (1381-2523) | 817-5424 | 26 | 2187  (717) | 2281  (1757-2412) | 666-3913 |
| **B-cells (CD19)** | 26 | 274  (235) | 227  (135-315) | 50-1164 | 26 | 277  (139) | 272  (186-320) | 44-638 |
| **NK-cells** | 26 | 255  (126) | 237  (190-288) | 80-563 | 26 | 248  (144) | 217  (141-343) | 29-647 |
| **T-cells (CD3)** | 26 | 1454  (838) | 1163  (958-1743) | 500-4607 | 26 | 1650  (569) | 1646  (1362-1943) | 548-3115 |
| **CD4** | 26 | 1001  (667) | 787  (646-1295) | 282-3482 | 26 | 1111  (431) | 1140  (783-1357) | 269-2068 |
| **CD4 Naive** | 26 | 391  (422) | 290  (191-414) | 55-2141 | 26 | 431  (252) | 384  (219-619) | 29-941 |
| **CD4 CM** | 26 | 504  (285) | 474  (303-615) | 9-1253 | 26 | 584  (230) | 585  (419-724) | 151-1106 |
| **CD4 EM** | 26 | 82.7  (60.1) | 66.1  (44.6-102.8) | 0.8-210.4 | 26 | 75.7  (37.5) | 79.3  (49.3-107.2) | 18-151.5 |
| **CD4 TEMRA** | 26 | 23.0  (38.7) | 3.6  (1.5-29.8) | 0.6-165.7 | 26 | 20.7  (30.8) | 5.8  (2.1-23.1) | 0.8-117.9 |
| **% CD69+CD4+** | 20 | 13.4  (9.2) | 10.9  (9.0-13.5) | 5.3-43.7 | 21 | 10.4  (2.6) | 11.1  (8.3-12.3) | 5-13.7 |
| **CD8** | 26 | 415  (245) | 342  (246-496) | 35-980 | 26 | 497  (213) | 475  (354-630) | 179-1025 |
| **CD8 Naive** | 26 | 106.6  (97.8) | 84.3  (46.2-137.9) | 12-510 | 26 | 139.4  (100) | 107.3  (66.5-184.4) | 16-457 |
| **CD8 CM** | 26 | 78.2  (54.2) | 69.2  (34-107.6) | 0.8-234.5 | 26 | 95.9  (49.4) | 78.5  (59.5-122.9) | 33.6-216.6 |
| **CD8 EM** | 26 | 51.7  (42.3) | 35.7  (24.1-71.2) | 0.8-162.2 | 26 | 65.5  (54.0) | 46.9  (27.8-83.6) | 11.8-240.7 |
| **CD8 TEMRA** | 26 | 179  (159) | 164  (55-293) | 2-694 | 26 | 197  (138) | 163  (90-244) | 34-639 |
| **% CD69+CD8+** | 20 | 22.3  (16.6) | 17.4  (13.5-22.5) | 9.4-86.2) | 21 | 16.5  (4.3) | 15.4  (14.5-17.7) | 9.5-27.1 |

**Suppl. Table 4g: Lymphocyte counts and sub-cell types at 14 days by arm**

|  | **At 14 days** | | | | | | | |
| --- | --- | --- | --- | --- | --- | --- | --- | --- |
|  | **Control** | | | | **Ciclosporin** | | | |
| **Outcome** | **n** | **Mean (SD)** | **Median (IQR)** | **Range** | **n** | **Mean (SD)** | **Median (IQR)** | **Range** |
| **Lymphocytes (total) (CD45)** | 8 | 2322  (1292) | 2058  (1518- 2528) | 1129-5242 | 9 | 1987  (489) | 1937  (1854- 2018) | 1116-2726 |
| **B-cells (CD19)** | 8 | 213  (128) | 188  (115- 331) | 44- 393 | 9 | 203  (82) | 201  (178- 215) | 53- 349 |
| **NK-cells** | 8 | 382  (248) | 330  (209- 482) | 115- 898 | 9 | 267  (146) | 294  (220- 394) | 32- 447 |
| **T-cells (CD3)** | 8 | 1717  (1187) | 1405  (1170- 1587) | 836- 4572 | 9 | 1509  (414) | 1517  (1278- 1601) | 839- 2157 |
| **CD4** | 8 | 1200  (956) | 894  (743 | 605- 3525 | 9 | 1051  (360) | 984  (861- 1220) | 408- 1647 |
| **CD4 Naive** | 7 | 318  (121) | 346  (205- 410) | 138- 483 | 7 | 422  (209) | 430  (271- 633) | 78- 670 |
| **CD4 CM** | 7 | 471  (162) | 387  (343- 613) | 313- 730 | 7 | 483  (124) | 533  (428- 561) | 228- 590 |
| **CD4 EM** | 7 | 67.1  (27.2) | 69.6  (56.2- 73.6) | 19.3- 111.4 | 7 | 73.2  (37.8) | 86.8  (24.4- 98) | 19.7- 121.3 |
| **CD4 TEMRA** | 7 | 11.7  (16.6) | 2.8  (1.2- 23.5) | 1.1- 44.8 | 7 | 15.4  (29.2) | 4.9  (2- 7.7) | 1.2- 81.4 |
| **% CD69+CD4+** | 6 | 11.3  (0.9) | 11.3  (10.5- 11.7) | 10.2- 12.8 | 6 | 14.4  (2.6) | 14.4  (12.1- 17.2) | 11- 17.3 |
| **CD8** | 8 | 458  (252) | 462  (215- 600) | 194- 913 | 9 | 425  (143) | 393  (350- 515) | 253- 698 |
| **CD8 Naive** | 7 | 65.9  (33.9) | 51.1  (38.4- 92.8) | 33.9- 123.8 | 7 | 128.1  (80.9) | 90.8  (80.1- 148.8) | 63.2- 298.2 |
| **CD8 CM** | 7 | 81.7  (63.3) | 51.7  (31.2- 151.2) | 31- 190.6 | 7 | 82.3  (31.2) | 77.9  (58.1- 92.8) | 48.4- 144.2 |
| **CD8 EM** | 7 | 50.2  (25.2) | 38.9  (33.8- 78.5) | 18.2- 87.9 | 7 | 75.5  (43.3) | 64.6  (25.1- 116.4) | 25- 127.6 |
| **CD8 TEMRA** | 7 | 195  (159) | 137  (72- 398) | 35- 426 | 7 | 169  (92) | 147  (95- 291) | 70- 303 |
| **% CD69+CD8+** | 6 | 20.4  (3.9) | 20.0  (17.5- 20.9) | 16.6- 27.7 | 6 | 21.8  (6.4) | 18.8  (17.1- 26.8) | 16.9- 32.6 |

**Suppl. Table 5: Multiple linear regression showing continuous secondary outcomes between** arms

|  | Unadj. | **Control** | | **Ciclosporin** | | Difference |
| --- | --- | --- | --- | --- | --- | --- |
| Outcome | t-test  (p value) | n | Mean^a^  (SD) | n | Mean^a^  (SD) | Adj. diff in means^b^  (B-A) (95%CI) |
| T lymphocyte counts (CD3) at:  5 mins  15 mins  30 mins  90 mins | 0.07  0.08  0.45  0.92 | 26  26  26  26 | 963  (534)  888  (522)  886  (632)  867  (572) | 26  26  26  26 | 1248  (565)  1190  (685)  1006  (506)  852  (472) | 142.4 (23.9 to 260.9)  150.1 (-48.6 to 348.8)  -23.7 (-192.3 to 144.8)  -106.5 (-331.4 to 118.4) |

^a^ univariate analysis reporting mean and SD at snapshot in time without adjustment

^b^ multivariate analysis reporting difference in means between groups at snapshot in time with adjustment for baseline measurement (0 mins) and stratification variables (ECG infarct location, gender and time between symptom onset and randomisation (0-3 hrs vs. 3-6 hrs))

**Suppl. Table 6: Multiple linear regression showing post-hoc continuous secondary outcomes between arms at 24 hours**

|  | Unadj. | **Control** | | **Ciclosporin** | | Difference |
| --- | --- | --- | --- | --- | --- | --- |
| Outcome at 24 hours | t-test  (p value) | n | Mean^a^  (SD) | n | Mean^a^  (SD) | Adj. diff in means^b^  (B-A) (95%CI) |
| B-cells (CD19)  NK-cells  T lymphocyte (CD3)  CD4  CD8 | 0.95  0.85  0.33  0.48  0.20 | 26  26  26  26  26 | 274 (235)  255 (126)  1454 (838)  1001 (667)  415 (245) | 26  26  26  26  26 | 277 (139)  248 (144)  1650 (569)  1111 (431)  497 (213) | 32.1 (-22.5 to 86.7)  -29.9 (-103.5 to 43.8)  57.4 (-264.3 to 379.0)  106.8 (-59.7 to 273.2)  32.0 (-92.9 to 156.9) |

^a^ univariate analysis reporting mean and SD at 24 hours without adjustment

^b^ multivariate analysis reporting difference in means between groups at 24 hours with adjustment for baseline measurement (0 mins) and stratification variables (ECG infarct location, gender and time between symptom onset and randomisation (0-3 hrs vs. 3-6 hrs))
