# Supplementary material for: Effect of ciclosporin on safety, lymphocyte kinetics and left ventricular remodelling in acute myocardial infarction
Source: Br J Clin Pharmacol. 2020 Mar 11;86(7):1387–97. doi: 10.1111/bcp.14252 (PMC7318996; doi:10.1111/bcp.14252)
Supplement: Supplementary file 2 — Data S1. Supporting Info Item [file BCP-86-1387-s002.docx]

**Supplemental Methods**

# **List of abbreviations**

| **Abbreviation** | **Meaning** |
| --- | --- |
| **APC** | Allophycocyanin |
| **BD** | Becton, Dickinson and Company |
| **CCR7** | CD197 |
| **CD4** | Cluster of differentiation 4 |
| **CD8** | Cluster of differentiation 8 |
| **FITC** | Fluorescein isothiocyanate |
| **FMO** | Fluorescence minus one |
| **MFI** | Mean fluorescence intensity |
| **PE** | Phycoerythrin |
| **STEMI** | ST-segment elevation myocardial infarction |
| **TCM** | Central memory T-cell |
| **TEM** | Effector memory T-cell |
| **TEMRA** | CD45RA+ effector memory T-cell |
| **TIMI** | Thrombolysis in myocardial infarction |
| **TNaïve** | Naïve T-cell |

#

**Study population**

All participants had to present with an acute myocardial infarction (STEMI) and undergo primary percutaneous coronary intervention (primary PCI), be at least 18 years of age, and present within 6 hours of the onset chest pain and ST segment elevation. The culprit coronary artery had to be a major coronary artery with a diameter of at least 3 mm and had to be proximally occluded (TIMI flow grade 0-1) at the time of admission coronary angiography.

Exclusion criteria for this trial were patients presenting with immunological disorders, cardiogenic shock, unconscious patients, evidence of coronary collaterals to the infarct area, open (TIMI>1) culprit coronary artery at the time of angiography, previous myocardial infarction or thrombolytic therapy, known renal or liver insufficiency, uncontrolled hypertension (>180/110 mmHg), female patients currently pregnant or women of childbearing age who were not using contraception (verbal diagnosis), or patients with contraindication to cardiac MRI.

**PCI and study treatment**

Patients underwent primary PCI according to standard guidelines. All patients received a loading dose of two antiplatelet drugs (see Table 2), and heparin before PCI. Use of thrombus aspiration and/or glycoprotein IIb/IIIa inhibition was left to the discretion of the treating physician. Zotarolimus- or everolimus-eluting stents were used.

**Experimental protocol**

Following angiography participants were randomised in a 1:1 ratio to either ciclosporin or control (saline) using a blocked allocation (permuted random blocks of variable length) system. Randomisation included stratification by infarct location (anterior or non-anterior) and gender. After the first 31 patients gender was replaced by ‘time from symptom onset to randomisation’ (0-<3 hours or 3-≤6 hours). This was implemented using using Newcastle Clinical Trials Unit’s online randomisation service. Ciclosporin (Sandimmun®, Novartis) was given as an intravenous infusion dissolved in saline (maximum concentration 2.5mg per millilitre) of 2.5mg per kilogram of body weight (maximum total amount 250 mg) through a catheter positioned within a peripheral vein over 4 min. Trial medication was prepared by two unblinded, GCP-trained research nurses according to local pharmacy policy. Due to the colour of the drug (concentrate for infusion could be clear to brown-yellow in colour), the infusion bag was covered and dark coloured lines were used to maintain the blinding of the operating clinician and patient. After successful infusion, the coronary wire was advanced and culprit lesion crossed, followed by revascularisation.

Primary outcome measure was infarct size at 12 weeks post-PPCI as measured by cardiac magnetic resonance imaging (MRI). Infarct size was calculated as the percent of infarcted myocardium per left ventricular (LV) mass.

Secondary outcome measures included cardiac MRI (EDV, ESV, SV, LVEF, myocardial mass (diastole/systole)), late gadolinium enhancement outcomes at 2-7 days (baseline) and 12 weeks (microvascular obstruction at baseline only, infarct size, myocardial mass), change in T lymphocyte counts (including CD45, B-cells (CD19), NK-cells, T-cells (CD3) and CD4 and CD8 subtypes) relative to baseline at 5, 15, 30,90 minutes and 24 hours post-reperfusion. Additionally, we also reported the number of clinical events (all cause death, stroke or myocardial infarction) after 12 months. We additionally reported left ventricular remodelling, kinetics of lymphocyte populations and additional T-cell subpopulations and selected cytokines in post-hoc analyses.

**Cardiac Magnetic Resonance Imaging**

Cardiac magnetic resonance scans were obtained at 2-7 days as well as 12 weeks post myocardial infarction with a Siemens Avanto 1.5 Tesla MRI scanner, using a phased array body coil combined with a spine coil. Intravenous Gadobutrol contrast (Gadovist, Bayer Schering Pharma AG, Berlin, Germany) was administered at a dose of 0.1mmol/kg, and after 10 minutes short axis end-diastolic LGE (late gadolinium enhancement) images were obtained. All analysis was performed using validated cardiac MRI analysis software (cvi42, Circle Cardiovascular Imaging Inc., Calgary, Canada) as previously described (Schwinzer & Siefken, 1996). The primary endpoint, percentage infarct size post-PPCI (as measured by cardiac MRI) was compared at 12 weeks between groups using the two-sample t-test. This analysis was then repeated using multiple linear regression with adjustment for all stratification variables used during the course of the trial (infarct location (anterior or non-anterior), gender and time from symptom onset to randomisation (0 to <3 hours or 3 to ≤6 hours)).

**Blood Tests**

Serum creatinine and ciclosporin serum levels were measured by the Freeman Hospital Clinical Biochemistry department. IP-10/CXCL10 serum levels were determined by using the Quantikine ELISA from R&D Systems, Minneapolis, USA.

**Flow Cytometry**

Upon arrival, EDTA blood samples were immediately placed on a roller mixer, and kept in the dark at room temperature. All cell populations were quantified as previously published (Boag *et al.*, 2015). Four sub-populations of CD4^+^ and CD8^+^ T-cells were investigated: naïve (T_Naive_), central memory (T_CM_), effector memory (T_EM_) and terminally differentiated effector memory cells (T_EMRA_), as previously described (Koch *et al.*, 2008).

**Statistical Analysis**

The planned sample size was 68 (34 in each arm) to allow 60 patients to complete the trial based on an anticipated loss to follow-up rate of 10%. This was decided pragmatically based on potential time available for recruitment and is the minimum conventional threshold for making parameter estimates in pilot studies (Lancaster *et al.*, 2004). Descriptive statistics are reported along with the appropriate confidence interval or standard deviation. The primary outcome is compared between trial arms using the 2-sample t-test. In addition, more exploratory analyses using multiple linear regression adjusting for stratification factors were undertaken. Owing to the relatively small sample size, more extensive modelling was not performed. Analysis of secondary outcome measures followed the same methods as for the primary outcome. Group membership was defined on an intention-to-treat basis. The extent of missing data is described implicitly by reporting of the number of participants available for each analysis. As a result of the small sample size, there was no use of any imputation techniques.

**Flow cytometry assays**

Four different assays were used for flow cytometric analysis of the blood samples collected. The 4-colour and 6-colour assays involved the use of fluorescence minus one (FMO) control samples; this enabled accurate gating of the positive population. Upon arrival, samples were immediately placed on a Stuart® roller mixer SRT6, ensuring sufficient mixing, and kept in the dark at room temperature. Incubation phases for all assays were carried out in the dark at room temperature. Analysis was performed by a research fellow (S.C.).

###

### TruCount

The absolute cell count of the various T-cell subpopulations was measured using a TruCount assay. 50μL fresh blood aliquots from each time point was added to their corresponding BD TruCount tubes (340334, BD Biosciences) using an eLINE Electronic 1-channel Pipette (15005787, Sartorius). Extra caution was exercised in ensuring that the tip is clean and rid of any blood as this could affect the absolute count of the cell populations. 10μL of BD Multitest 6-Color TBNK (337166, BD Biosciences) was deposited on the side of the tube, ensuring no contact was made with the sample. The BD Multitest 6-Color TBNK consisted of CD3 Fluorescein isothiocyanate (FITC), CD16 Phycoerythrin (PE), CD56-PE, CD45-Per-Cy5.5, CD4-PE-Cy7, CD19 Allophycocyanin (APC) and CD8-APC-Cy7 in buffer with 0.1% sodium azide. After vortexing the test sample using a Vortex Genie 2 (Scientific industries), the mixture was incubated before achieving red blood cell lysis by adding 1.5mL of fresh lysis buffer prepared using BD Pharm Lyse Lysing Buffer (10x Conc. 5075567, BD Biosciences) and Gibco distilled water (15230-147, Life technologies). After another period of incubation, the samples were run through a BD FACSCanto II Flow Cytometer (339473, BD Biosciences) using the BD FACSCanto acquisition software, which was set up using BD FACS 7-color Setup Beads (335775, BD Biosciences) and BD FACS Setup Bead Diluent (336565, BD Biosciences).

**Suppl. Figure 1** displays the gating strategy used for the TruCount assay.


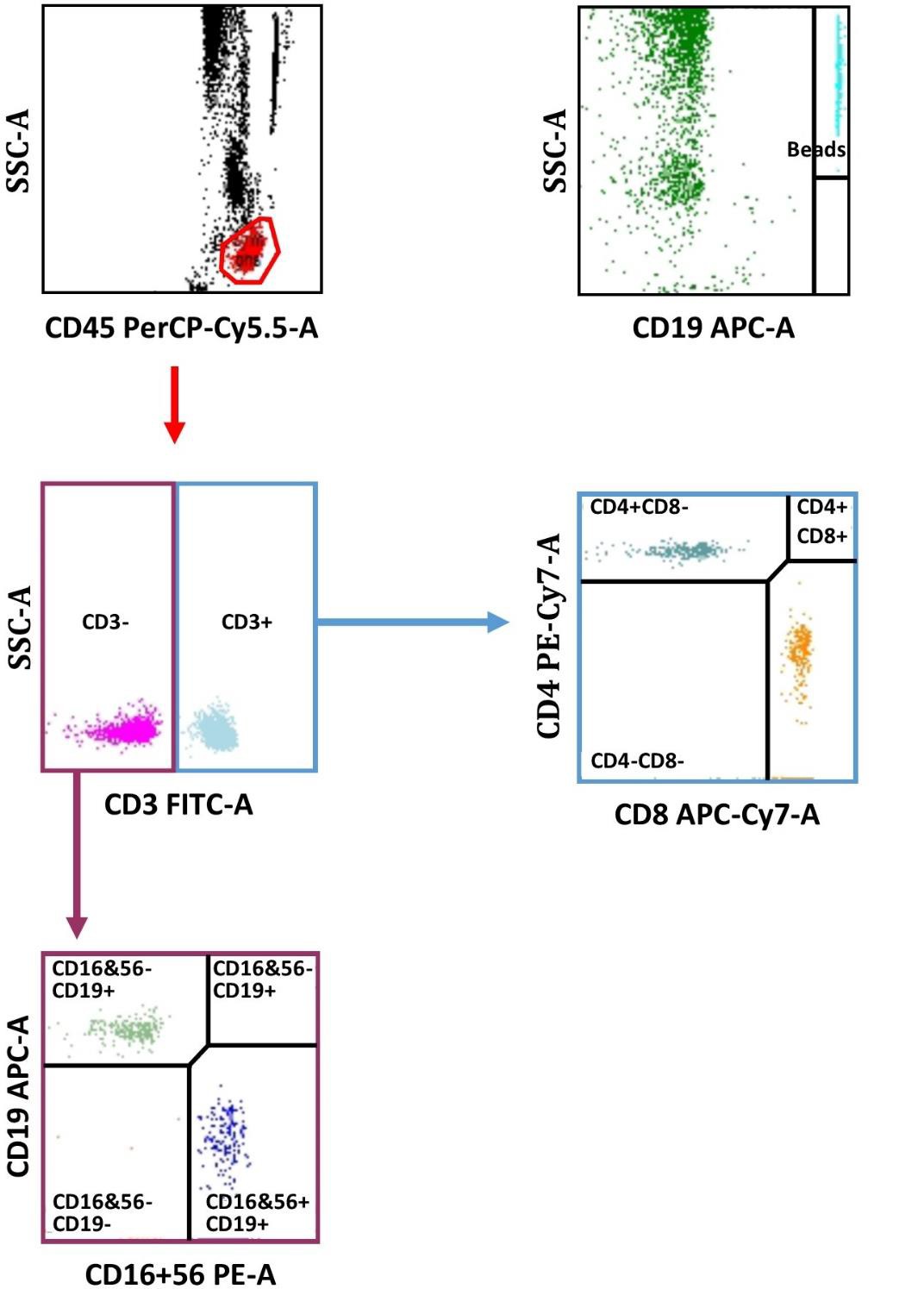


**Suppl. Figure 1**. TruCount assay gating strategy. Lymphocytes were further gated into CD3+ T-cells and CD3- lymphocytes. The latter consisted of either Natural Killer cells (CD3-CD19-CD16/56+ cells) or B-lymphocytes (CD19+). The addition of a known number of hyper-fluorescent TruCount beads in a known volume of fresh blood allows for quantification per volume of blood.

### 4-colour assay

T-cell activation was assessed using a 4-colour flow cytometric assay. 50μL fresh blood aliquots from the PRE, 90 minute and 24-48 hour samples was added to their corresponding labelled 5mL Falcon® Polystyrene Round-Bottom tubes. A cocktail of antibodies, detailed in **Table 1**, were added to the sample and allowed to incubate for 20 minutes. 1mL of fresh lysis buffer, was added to each mixture. Following another 20 minutes of incubation, the sample was washed two times using a BD FACS-II Lyse/Wash Assistant (337146, BD Biosciences). The washed sample was then run through a BD FACSCanto-II Flow Cytometer (339473, BD Biosciences) using the BD FACSDiva acquisition software. Cells were gated on CD3+ and 20,000 events were recorded allowing for representative data. The 4-colour assay gating strategy is shown in **Suppl. Figure 2.**

| **Antibody** | **Catalogue Number** | **Manufacturer** |
| --- | --- | --- |
| BD Pharmingen PE Mouse Anti-Human CD3 | 555333 | BD Biosciences |
| BD Horizon V500 Mouse Anti-Human CD4 | 560768 | BD Biosciences |
| BD Pharmingen FITC Mouse Anti-Human CD8 | 555366 | BD Biosciences |
| CD69 APC | 340560 | BD Biosciences |

**Table 1**. Antibodies used for the 4-colour flow cytometric assay with their respective catalogue number and manufacturer.


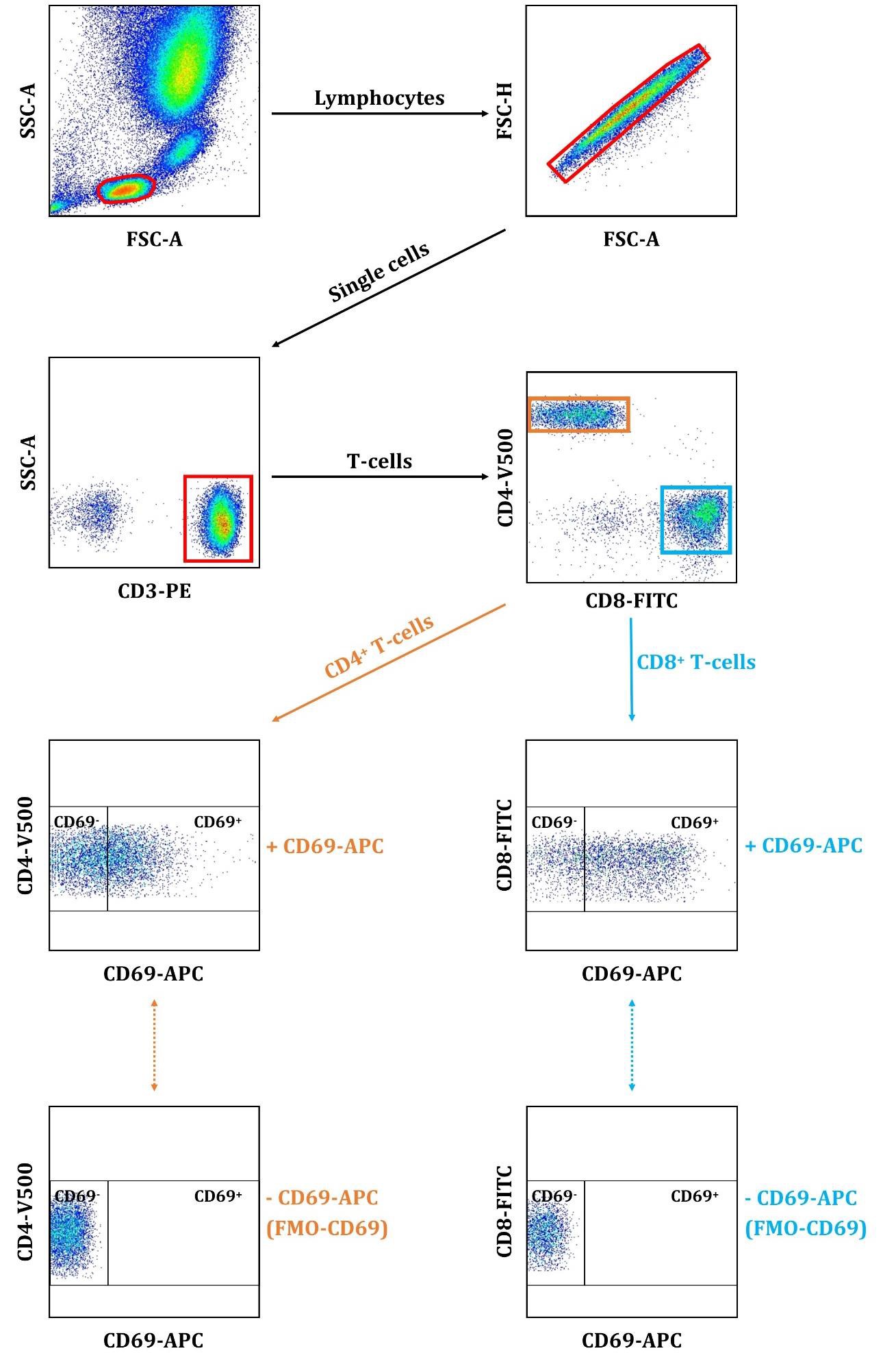


**Suppl. Figure 2**. 4-colour flow cytometry assay gating strategy. This assay was used to assess T-cell activation. FMO staining controls were used to determine the gating parameters for the CD69+ population.

**6-colour assay**

Four sub-populations of CD4^+^ and CD8^+^ T-cells were investigated: T_Naive_, T_CM_, T_EM_ and T_EMRA_ , as previously described (Koch *et al.*, 2008). 50μL fresh blood aliquots from every time point was added to their corresponding labelled 5mL Falcon tubes. A cocktail of antibodies, detailed in **Table 2** were added to the sample. After a 20 minute incubation, 1mL of fresh lysis buffer was added to each mixture. Following another 20 minute incubation, the sample was washed and run through a BD FACSCanto-II Flow Cytometer (339473, BD Biosciences) using the BD FACSDiva acquisition software. Stopping gate and events recorded were the same as that of the 4-colour assay. The 6-colour assay gating strategy is shown in **Suppl. Figure 3**.

| **Antibody** | **Catalogue Number** | **Manufacturer** |
| --- | --- | --- |
| BD Pharmingen PE Mouse Anti-Human CD3 | 555333 | BD Biosciences |
| BD Horizon V500 Mouse Anti-Human CD4 | 560768 | BD Biosciences |
| BD Pharmingen FITC Mouse Anti-Human CD8 | 555366 | BD Biosciences |
| Brilliant Violet 421 anti-human CD197 (CCR7) | 353208 | BioLegend® |
| CD45RA (L48) PE- Cy7 | 337186 | BD Biosciences |
| APC anti-human CX3CR1 | 341610 | BioLegend® |

**Table 2**. Antibodies used for the 6-colour flow cytometric assay with their respective catalogue number and manufacturer.


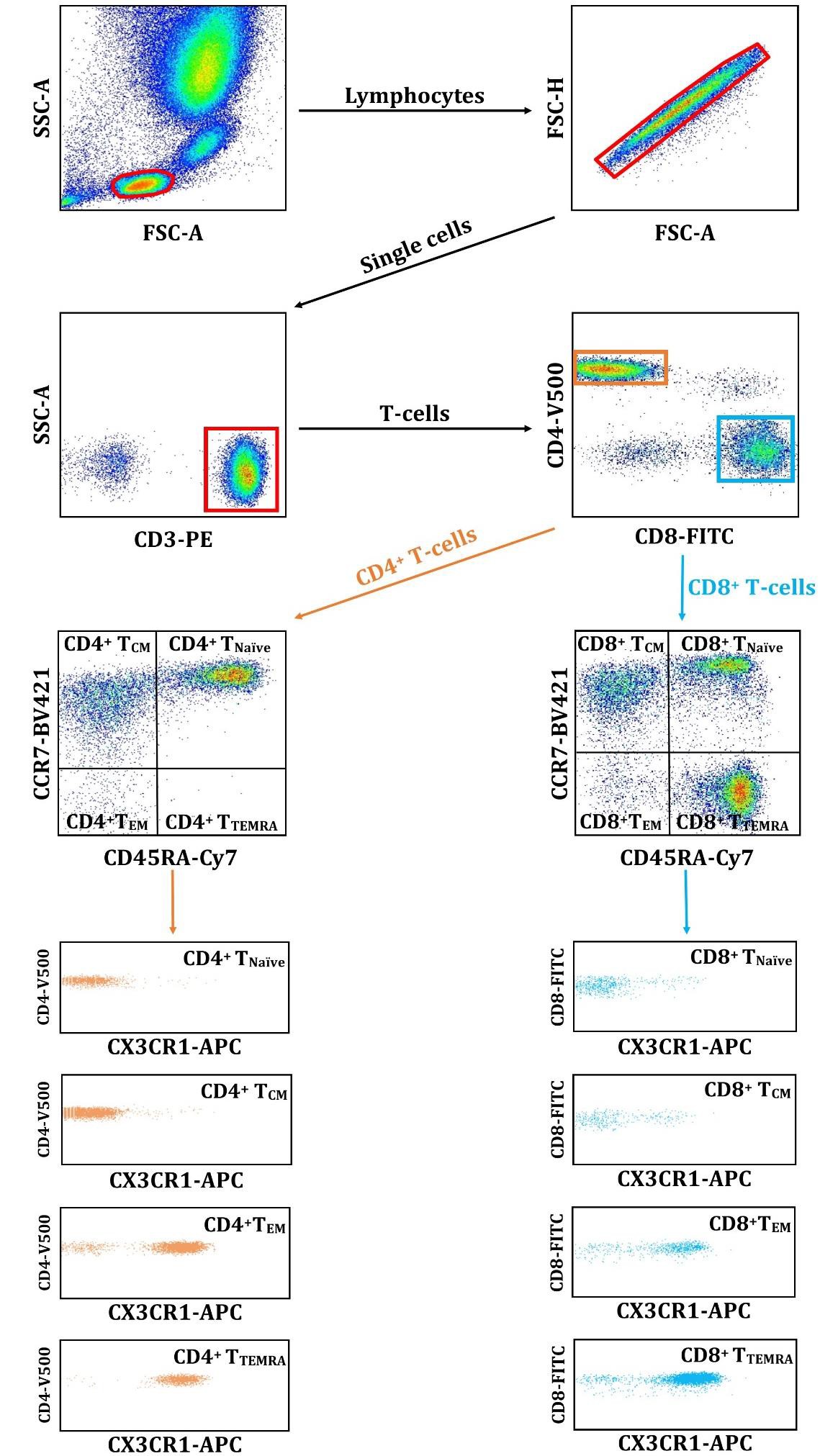


**Suppl. Figure 3**. 6-colour flow cytometry assay gating strategy. This assay was used to assess CX_3_CR1 expression and the relative numbers of each T-cell subpopulation. FMO staining controls were used to determine the gating parameters for the CD45+ and CCR7+ population.

## Cardiac MRI Image Acquisition:

Cardiac magnetic resonance (CMR) scans were obtained at 2-7 days post myocardial infarction (at an average of 3 days) with a Siemens Avanto 1.5 Telsa MRI scanner, using a phased array body coil combined with a spine coil. All images will be obtained during breath holding. Localiser images were acquired as well as axial black blood HASTE images to define anatomy. Cine images of the heart in 2, 3 and 4 chamber views were obtained using a steady state free precession pulse (SSFP) sequence (repetition time [TR]: set according to heart rate, image matrix 144x192, echo time (TE): 1.19ms, flip angle: 80°). T2 weighted STIR (short inversion time [TI] inversion recovery) images were then obtained in the same projections, using a black-blood segmented turbo spin echo technique (TR according to heart rate, TE 47ms, flip angle 180°, TI 140ms, image matrix 208x256). Further sequential end-diastolic STIR images were acquired along the short axis of the heart, covering the full extent of the left ventricle in parallel slices (each 8mm with 0mm gap). Corresponding short axis SSFP cine images were then obtained to allow quantification of chamber volumes and function. Intravenous Gadobutrol contrast (Gadovist, Bayer Schering Pharma AG, Berlin, Germany) was administered at a dose of 0.1mmol/kg, and after 10 minutes short axis end-diastolic LGE images (in corresponding locations to cine and STIR images) was obtained using an inversion recovery (IR) segmented gradient echo sequence (TR: according to heart rate, TE: 3.41ms, flip angle: 25°, image matrix: 196x256). The inversion time (TI) for LGE imaging was selected in order to null normal myocardium (giving it a dark appearance), and adjusted throughout acquisition (increased approximately every second slice) to maintain nulling.

## Cardiac MRI Image Analysis

All analysis was performed using validated cardiac MRI analysis software (cvi42, Circle Cardiovascular Imaging Inc., Calgary, Canada). The analysis was performed by the research fellow (A.M.) and validated by cardiac imaging consultant (R.D), following formal training in CMR analysis and cvi42 software. In order to prevent bias, analysis was performed in batches, using anonymised images, which are subsequently linked back to the relevant clinical/FACS data. LV volumes and mass was determined using the short axis SSFP cine images, following determination of the longitudinal extent of the chamber by cross referencing with the 4 and 2 chamber images, as previously described and validated [40]. Epicardial and endocardial borders were traced automatically on each end-systolic and end-diastolic short axis cine frame with manual correction where necessary, allowing automated calculation of left ventricular mass, dimensions and ejection fraction (LVEF) (Suppl. Figure 4).

**Suppl. Figure 4. LV mass and volume assessment by CMR**

**A**: Basal ventricular short axis slice at end diastole showing endocardial border (red), epicardial border (green) **B**: Corresponding long axis reference image in both 2 and 4 chamber views, showing the slice position (highlighted in yellow). **C**: Equivalent short axis slice (**C**) and long axis reference images (**D**) at end systole.

In order to quantify infarct size and MVO, the short axis LGE images was used, all of which were taken at end diastole. Epicardial and endocardial borders were then traced on each LGE slice, and a reference region of normal myocardium identified using an automated method with manual correction where necessary. Areas of enhancement (infarction) was identified and quantified automatically using a signal intensity threshold of 5 standard deviations above normal remote myocardium, as previously described and validated [41]. Regions of hypoenhancement within the enhanced zone (microvascular obstruction – MVO), were identified and quantified using semi-automatic thresholding following manual border delineation of areas of interest, and included in the calculated infarct mass (Suppl. Figure 5).

**Suppl. Figure 5. Analysis of LGE images for infarct size and MVO quantification**

**A+B:** Short axis LGE image showing inferior infarct. **A:** Raw image without analysis, in which normal myocardium appears dark and infarct zone shows enhanced (white) appearance **B:** Corresponding analysed image showing myocardial borders (red: endocardial, green: epicardial) as well as normal myocardium reference area (blue border) and region of enhancement (infarct, yellow shading). **C+D**: Short axis LGE images showing anteroseptal infarct with extensive MVO **C:** Raw unanalysed image in which dark core of MVO can be clearly seen within hyperenhanced infarct. **D** Corresponding analysed image shows all contours and analysis, with infarct area shown in yellow and MVO shaded orange.

References

Boag, SE, Das, R, Shmeleva, EV, Bagnall, A, Egred, M, Howard, N, Bennaceur, K, Zaman, A, Keavney, B, Spyridopoulos, I (2015) T lymphocytes and fractalkine contribute to myocardial ischemia/reperfusion injury in patients. *J Clin Invest* **125**, 3063-3076.

Koch, S, Larbi, A, Derhovanessian, E, Ozcelik, D, Naumova, E, Pawelec, G (2008) Multiparameter flow cytometric analysis of CD4 and CD8 T cell subsets in young and old people. *Immun Ageing* **5**, 6.

Lancaster, GA, Dodd, S, Williamson, PR (2004) Design and analysis of pilot studies: recommendations for good practice. *J Eval Clin Pract* **10**, 307-312.

Schwinzer, R, Siefken, R (1996) CD45RA+ and CD45RO+ T cells differ in susceptibility to cyclosporin A mediated inhibition of interleukin-2 production. *Transpl Immunol* **4**, 61-63.
